# Novel nonthermal food processing practices: Their influences on nutritional and technological characteristics of cereal proteins

**DOI:** 10.1002/fsn3.2792

**Published:** 2022-02-28

**Authors:** Neda Mollakhalili‐Meybodi, Roghayeh Nejati, Mehran Sayadi, Amene Nematollahi

**Affiliations:** ^1^ Department of Food Sciences and Technology School of Public Health Shahid Sadoughi University of Medical Sciences Yazd Iran; ^2^ Research Center for Food Hygiene and Safety Shahid Sadoughi University of Medical Sciences Yazd Iran; ^3^ Department of Food Safety and Hygiene School of Health Fasa University of Medical Sciences Fasa Iran

**Keywords:** cereal, nonthermal processing, protein

## Abstract

Cereals, as the main crops cultivated and consumed in the world, are a rich source of carbohydrates, proteins, dietary fiber, and minerals. Despite the nutritional importance, their technological applicability in food matrices is also considerably important to be determined. Cereal processing is done to achieve goals as increasing the shelf‐life, obtaining the desired technological function, and enhancing the nutritional value. Nonthermal processing is preferred regarding its potential to provide beneficial impacts with minimum adverse effect. Technological functionality and nutritional performance are considered as the most basic challenges through cereal processing, with proteins as the main factor to take part in such roles. Technological and nutritional functionalities of proteins have been found to be changed through nonthermal processing, which is generally attributed to conformational and structural changes. Therefore, this study is aimed to investigate the impact of nonthermal processing on nutritional and technological characteristics of cereal proteins.

## Graphical abstract

- Several nonthermal techniques have been applied for cereal processing with minimum effect on their quality characteristics
- These methods improve the nutritional and functional properties of cereal proteins
- They are an excellent alternative to detrimental thermal food processing techniques
- There are several cereals’ nonthermal processing techniques. These methods improve the nutritional and functional properties of cereal proteins. They have an impact on the protein structure and consequently the conformational changes. These nonthermal processing methods usually have a minimum effect on cereals’ quality characteristics. They are an excellent alternative to detrimental thermal food processing techniques.

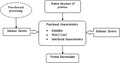

## 1 INTRODUCTION

Cereals are one of the main food crops in the world, full of carbohydrates, proteins, dietary fiber, and minerals. Wheat, corn, rice, and barley are the most popular cereal grains regarding production quantity. Other cereal grains including sorghum, rye, oat, and millets are produced in small quantities. According to the Food and Agriculture Organization (FAO), the global demand for wheat, corn, and rice is expected to reach 3.3 billion tons per year by 2050, due to the rapid population growth (Guerrieri & Cavaletto, 2018). Cereals are mostly used as processed products viz breads, noodles, pasta, breakfast cereals, snack foods, cookies, etc. Cereal processing is one of the most important technologies in the food system to achieve many goals such as increasing shelf‐life, obtaining the desired flavors, and enhancing the nutritional value (Sasthri et al., 2021). Technological properties and nutritional quality are among the most basic challenges in cereal processing, which is effectively influenced by grain proteins (Joye, 2019).

The protein content of cereals is an average of 7%–15% by weight, which is distributed nonuniformly in different parts of the whole grain (Guerrieri & Cavaletto, 2018). The total content and composition of proteins in cereals are widely influenced by genotype (species, varieties) and plant growth conditions (climate, soil, fertilization) (Koehler & Wieser, 2013). Despite the low quality and quantity (Guerrieri & Cavaletto, 2018), allergenic (Scherf et al., 2016), and antinutritional effects (Kostekli & Karakaya, 2017), cereal proteins can be considered as the main sources of dietary protein due to the availability of cereals as a main cheap foodstuff (Guerrieri & Cavaletto, 2018). For long times, traditional thermal methods have been used in cereal grains and their products with the aim of microorganisms’ removal and increasing shelf‐life (Ozkan et al., 2019). The thermal processing induces changes in the structure of food macromolecules, including proteins, which is revealed by denaturation and related reactions (Sasthri et al., 2021). These alterations in cereal proteins are followed by benefits such as increasing digestibility (Raghuvanshi et al., 2011), reducing allergenicity (Ekezie et al., 2017), inactivation of antinutrients (Joye, 2019), and providing the desired aroma, flavor, and color (Helou et al., 2016). Despite the benefits of traditional thermal processing, undesirable effects of these methods, especially on the nutritional quality of food, are well known. For instance, heating reduces the nutritional quality of cereal proteins by promoting the racemization reactions (Gilani et al., 2012), production of undesirable compounds (Çelik & Vural, 2020), formation of disulfide (Annor et al., 2017) and isopeptide bonds (Joye, 2019). These adverse effects have led to intensified researches into the development of nonthermal methods in the recent decades (Picart‐Palmade et al., 2019). Nonthermal methods make it possible to increase food safety and shelf‐life with minimal changes in nutritional and functional characteristics. Several nonthermal techniques, which have gained popularity for cereal processing, include cold plasma (CP), high hydrostatic pressure (HHP), ionizing irradiation, pulsed electric field (PEF), pulsed light (PL), and ultrasonic (US).

These methods also improve the nutritional and functional properties of cereal proteins by affecting their structure. Results of cereal processing by nonthermal methods confirmed the reduction of allergenicity of wheat proteins (Nooji, 2011), increasing the digestibility of corn flour (Hassan et al., 2009), promoting the bioactivity of brown rice proteins (Lee & Kim, 2018), improving dough strength (Misra et al., 2015), enhancing the solubility of rice proteins (Zhang et al., 2018), and increasing emulsion stability of wheat flour (Bhat et al., 2016). To the best of our knowledge, there is no review about the influences of emerging nonthermal technologies on nutritional and technological characteristics of cereal proteins. With regard to the importance of cereal proteins in the diet and the advantages of the noted nonthermal methods, the aim of this study was to review the effects of these methods on the cereal proteins regarding their technological properties and nutritional characteristics. Furthermore, we discussed the principles of nonthermal technologies in the food processing.

## 2 PRINCIPLE OF NOVEL NONTHERMAL FOOD PROCESSING

**FIGURE 1:**
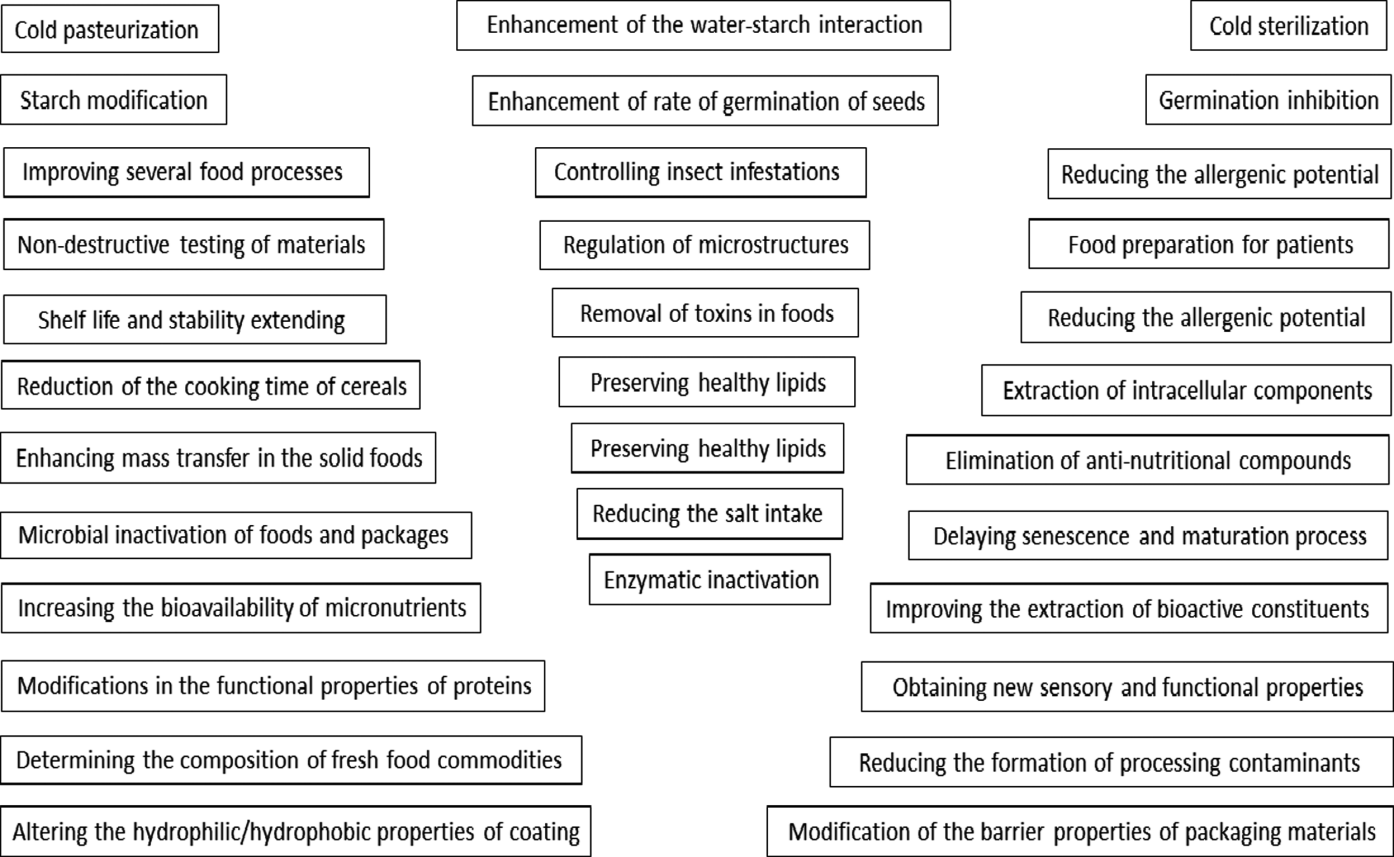
Main applications of novel nonthermal technologies in the food industries

Nonthermal procedures (NTPs) are novel and promising technologies which have gained considerable attention in the recent years. Regarding their preservation and maintenance, with minimum effect on the quality characteristics of foods, NTPs are known as potential alternatives to conventional thermal procedures (López et al., 2019). They have been used in the food safety sector to achieve different desired outcomes including decontamination of food products and modification of functional properties of food components like proteins and starch. All these changes are achieved without causing noticeable loss in nutritive and sensory attributes of foods (Zhang et al., 2019). In this section, the principles, functions as well as advantages and disadvantages of the most important nonthermal technologies including CP, HPP (high pressure processing), ionizing irradiation, PEF, PL, and US will be discussed one by one. Figure 1 shows the main applications and impacts of these technologies in the food safety and quality sectors.

### 2.1 Cold plasma

Cold plasma (CP), as a novel nonthermal technology, comprises the creation of extremely reactive ionized gases, that is, plasma, by intensifying their energy quantities and directly utilizing them to process food for specific purposes (Adebo et al., 2020a). Plasma, regarded as fourth state of matter, is the prevalent state within the universe (Mollakhalili‐Meybodi et al., 2021). Upon increasing the energy of a gas, plasma is generated (Feizollahi et al., 2020). The plasma structure involves a discharge machine, processing chamber, gas and pressure control systems (Zhang, Wang, Chen, et al., 2019). Several plasma sources have been developed including corona, dielectric barrier, and microwave discharge as well as gliding arc, and plasma jet (Tolouie et al., 2018). These systems can use different types of energy sources vis. thermal, electric, magnetic, microwave, and radio‐frequency (RF). It is worthy to note that electric and electromagnetic energy supplies are the most common procedures in plasma generation (Gavahian & Cullen, 2020). The plasma produced under low gas pressure and low power conditions is the cold plasma or nonequilibrium plasma which has low temperatures (Gavahian & Cullen, 2020). Plasma could be generated in any gas like oxygen, nitrogen, or noble gases such as helium, neon, and argon by giving enough energy able to initiate ionization of the gas (Yodpitak et al., 2019). In fact, plasma is an ionized gas consisting of ultraviolet (UV) photons, negative and positive ions, free radicals, excited or neutral atoms, and molecules as well as free electrons which could generate reactive species including reactive oxygen species (ROS) (like atomic oxygen, ozone, singlet oxygen, superoxide, and hydroxyl radicals) and reactive nitrogen species (like nitric oxide and nitrogen dioxide) during food processing (Zare et al., 2021). These reactive species are effective in the rapid microbial deactivation through oxidation of main cell components like DNA, proteins, and cytoplasmic membranes (Kaur et al., 2020). Thus, cold plasma processing could extend the shelf‐life and safety of food products at room temperature and atmospheric pressure conditions, without producing considerable perceivable alterations in food quality (Gavahian & Cullen, 2020). Along with the decontamination efficiency, cold plasma may also alter the structural properties of food components like proteins and starch to influence their functionality in cereal‐based products (Held et al., 2019). Actually, the noted reactive species present in cold plasma could break peptide bonds, oxidize amino acid side chains, generate cross‐links inside the proteins, and result in accumulation incident, which are preferred by the generation of intra‐ and intermolecular disulfide bonds. All these reactions lead to alterations in the configuration and three‐dimensional (3D) structure of proteins (López et al., 2019).

There are several advantages concerning the cold plasma procedure which renders it as a promising unique food technology including rapid processing times, plain design, simple application, effectiveness at low temperatures (suitable for heat‐sensitive products), economic and environmentally friendly procedure, nontoxic nature, and applicability for prepackaged foods. However, cold plasma has not been applied commercially until now in the food industry due to high capital investment (Kaur et al., 2020; López et al., 2019).

### 2.2 High pressure processing

High pressure processing (HPP), recognized as high‐hydrostatic pressure processing (HHPP) or ultra‐high pressure processing (UHPP), is one of the most important nonthermal food treatment technologies which subject foods to much higher pressures than atmospheric pressure to extend the shelf‐life of foods with minimal effects on their sensory and nutritional properties (Balakrishna et al., 2020). The pressure and temperature used in this novel technology vary from 100 to 1000 MPa and −20 to 90°C, respectively, for a controlled time (Kaur et al., 2019). It is permitted as a nonthermal pasteurization method by the Food and Drug Administration (FDA) due to efficient inactivation of a wide range of pathogenic and spoilage microorganisms and several enzymes, as well (Adebo et al., 2020a). It is noteworthy that HPP, unlike cold plasma, is one of the two main technologies (after microwave heating) used for commercial applications in the last years for different types of food products (Aguirre et al., 2018).

For HPP treatment, the food products are usually placed into a basket after vacuum packaging, which is then loaded into the treatment chamber. After closing the two openings, a high pressure pump along with a pressure intensifier is applied to produce adequate pressure on foods for a specific time. An increase in water temperature occurred with the range of 3–9°C/100 MP an increase in pressure. This quick rise in temperature during pressurization and following cooling upon decompression is very much suitable for a heat‐sensitive food product (Pérez‐Andrés et al., 2018).

HPP can impact on protein unfolding and subsequent denaturation, microorganisms, and the chemical reactions. In fact, in this treatment high‐weight compounds like proteins can be affected because they are formed by weak bonds and forces (van der Waals, hydrogen, and hydrostatic). However, low‐weight compounds like vitamins are naturally resistant to HPP conditions due to possessing strong covalent bonds (Pérez‐Andrés et al., 2018). Temperature, pressure, and holding time are the main processing parameters in the HHP treatment (Picart‐Palmade et al., 2019).

HPP has benefits compared to typical thermal treatments including improving food functionality, retention of micronutrients, flavor and aroma, efficient inactivation of microorganisms, substantial energy savings, homogeneity of processing, minimal energy needed, and negligible heat effect (Adebo et al., 2020b; Kaur et al., 2019).

### 2.3 Ionizing irradiation

Irradiation is a physical process which exposed foods to radiation of various frequencies and energies (Hernández‐Hernández et al., 2019). In fact, food irradiation is approved as an alternative to common thermal processing methods (Kim, Ramakrishnan, et al., 2020). Ionizing radiation commonly comprises gamma radiation, electron beams (accelerated electrons), and X‐rays. Gamma‐rays are produced using radionuclides like Cobalt 60 or Cesium 137 (Hernández‐Hernández et al., 2019). In electron beam irradiation (EBI), a focused beam of accelerated electrons (beta) is jetted on food products (Mohammadi Shad et al., 2019). X‐ray and electron beam technologies are formed from commercial electricity which makes them on–off equipment unlike gamma irradiation (Pan, Xing, Luo, et al., 2020). X‐rays are formed from machine supplies with energies close to 5 MeV, while accelerated electrons are generated from an electron accelerator with energies close to 10 MeV (Zhang, Wang, Chen, et al., 2019).

Ionizing irradiation has been applied for the food decontamination (insect and microbial removal) and modification of the physicochemical and functional characteristics of some components like proteins through free radical generation. Thus, this treatment could result in the improvement of food quality and enhancement of the shelf‐life (Kumar et al., 2017). Commonly, doses of 1–10 kGy are applied for pasteurization of perishable foods like fruits and vegetables, while doses of 10–50 kGy are used for sterilization of low‐moisture foods like cereals and spices (Ekezie et al., 2018). It is reported that irradiation doses of lower than 10 kGy are appropriate and safe for food decontamination without causing toxicity hazards which present only minimal nutrition effects (Pan et al., 2020).

The radiations could interact with different types of molecules to generate positively and negatively charged ions and/or excited species. These active free radicals show lethal effect to microorganisms and could also result in the modification of biomolecules including proteins and starch by destruction of covalent bonds. The effectiveness of this treatment is influenced by the nature of radiation, food composition, moisture level, oxygen content, the absorbed dose, and food dimensions (Hernández‐Hernández et al., 2019).

These types of nonthermal food treatment do not form radioactivity in food products and they are commercially available procedures. They could sustain food quality and obtain food safety without considerably affecting the organoleptic or nutritional characteristics (Kumar et al., 2020). There are other advantages regarding food irradiation including less treatment times, good penetration into foods, processing of packaged products, low energy charge, etc. However, this treatment procedure shows some disadvantages like high capital cost and low consumer acceptance (Hernández‐Hernández et al., 2019).

### 2.4 Pulsed electric field

Pulsed electric field (PEF) is one of the most superior nonthermal food preservation methods which contains utilization of short‐length electric pulses (1–100 μs) with high power into the food products placed between two electrodes producing high voltage pulses (20–80 kV/cm) (Ekezie et al., 2018). The PEF involves different systems viz the pulsed power, food movement, cooling, operating, or control as well as the treatment chamber (Zhang, Wang, Chen, et al., 2019). The treatment chamber is the main component of the PEF system, which can be worked in two different forms including static (batch) and continuous modes (Picart‐Palmade et al., 2019). In the static form, food is placed between two counterpart electrodes, while in the continuous form, food is shifted between the electrodes aided by a pump (Galván‐D’Alessandro & Carciochi, 2018).

High‐intensity PEF has made the conversion from the lab scale to the commercialized food sector with the main purpose of killing microorganisms (both pathogenic and spoilage types) mostly done by permeabilization and breakdown of cell membranes caused by electroporation (Yan et al., 2017). In fact, this phenomenon could result in pores’ generation within cellular membranes which cause cell inactivation. It is well known that using an electric discharge on a food, several chemically active species can be produced which eventually generate toxic chemical species, including hydroxyl radicals, chloride ions, and oxygen peroxide (Pérez‐Andrés et al., 2018).

The energy scattered during PEF treatment can also cause ionization of functional groups present in biomacromolecules. When it comes to proteins, this treatment could break electrostatic interactions in their chains leading to cleavage or combination of amino acids. Therefore, it is obvious that PEF could apply for the modification of proteins’ structure and their functional properties (Giteru et al., 2018).

Pulsed electric field has numerous advantages such as lower processing time and temperature, and lower energy consumption compared to conventional processing (Li et al., 2019). Furthermore, this technology shows uniform treatment intensity and continuous processing nature (Ahmed et al., 2020). Given the application of lower temperatures than 60°C in this process, PEF generally shows minimal alterations in nutritional and organoleptic properties of different food products compared to common thermal food processes. However, the industrial PEF equipment is limited due to high investment costs (Hernández‐Hernández et al., 2019).

### 2.5 Pulsed light

Pulsed light (PL) is also a promising nonthermal food treatment technology containing the utilization of extremely short high‐power electrical pulses of wide spectrum light (100–1100 nm) including 54% UV, 26% visible, and 20% infrared regions (Adebo et al., 2020a). Each pulse lasts a few seconds, although the power of each flash is 20,000 times higher than that of sunlight and involves some UV light. The main procedures that have been suggested for the effectiveness of PL are usually attributed to the UV fraction that includes photothermal and photochemical effects (Adebo et al., 2020b). Thus, the PL technique is also known as pulsed UV light which comprises a control unit to produce high‐intensity electrical pulses, a treatment chamber to convert the light source to high‐intensity light pulses as well as a timing control system and a start generator (Zhang, Wang, Chen, et al., 2019). Light‐emitting diode (LED) technique is an emerging procedure to produce and emit light pulses by electroluminescence resulted from electric current (Subedi et al., 2020).

In the PL system, high voltage is applied to motivate an inert gas such as xenon from a ground condition to an excited condition. Given the instability of xenon in this state, it liberates energy as photons to return to the ground state and subsequently the emitted energy is absorbed by the food components, causing sequences of photoreactions (thermal, physical, and chemical) in the food product (Ekezie et al., 2018). Given the presence of chromophores within the biomolecule like proteins, they are the main objects for photoreactions induced by PL. The absorption of pulsed UV light by the protein functional groups like aromatic amino acids (phenylalanine and tyrosine) results in the progress of chain oxidation, structure fragmentation, and creation of cross‐links and aggregates (Panozzo et al., 2016).

In fact, pulsed UV light is more effective in microbial killing than continuous UV light due to its excellent penetration ability and fast dissipation of high‐intensity pulses. The major potential advantages of PL include extremely short processing time, absence of toxic residues, simple adaptability, great flexibility, reduced energy prices, and high flexibility (Ekezie et al., 2018).

### 2.6 Ultrasonication

Ultrasonication (US) processing is another emerging nonthermal food treatment method which has recently attracted attention as an excellent alternative for common heat‐based technologies that are unfavorable for food product quality (Bhargava et al., 2020). US is considered as acoustic waves with frequencies that surpass the audible level of human perception (above 20 MHz) (Adebo et al., 2020b). It is actually generated by ultrasonic transducers that switch electrical energy into vibrational sound energy of required power. In fact, its principle is the scattering of sonic waves similar to light waves (Bhargava et al., 2020). The US system usually involves ultrasonic generator, oscilloscope, and treatment room (Zhao et al., 2019).

There are two types of US technologies based on the frequency, including high‐frequency US (low power) which is mainly used as a nondestructive diagnosis method and low‐frequency US (high power) which could have considerable effect on food properties (Pérez‐Andrés et al., 2018). This technology has several applications in the food industry including extraction, processing, and preservation. It is believed that high‐power US (with a frequency of 20–100 kHz and intensities of 10–1000 W/cm^2^) affects food quality via acoustic cavitation phenomena as well as mass transfer improvement, intense pressure, and strong shear (Bhargava et al., 2020). The waves occur in rotations of compression and expansion with adequate powers that could make cavitation bubbles. Across physicochemical procedures like diffusion, the cavitation‐created microbubbles’ size will quickly increase up to accomplishing a critical size at which it strongly collapses because of the failure to absorb energy. These conditions could considerably accelerate chemical activity and also cause strong hydrodynamic shear forces through the collapse of the microbubbles which are able to degrade large molecules like proteins which leads to mechanical and chemical alterations of the food matrix (Adebo et al., 2020b; Hernández‐Hernández et al., 2019). Furthermore, the free radicals produced by cavitation could trigger some reactions like lipid oxidation and protein denaturation (Bhargava et al., 2020).

Ultrasonic shows different advantages in food sectors, like greater product yield, better quality product, reduced temperature, decreased energy consumption, rapid processing times, minimal processing periods, decreased operating and protection prices, reduction of pathogens, increased the food shelf‐life, etc. (Bhargava et al., 2020).

## 3 THE INFLUENCE OF NOVEL NONTHERMAL FOOD PROCESSING PRACTICES ON TECHNOLOGICAL AND NUTRITIONAL CHARACTERISTICS OF CEREAL PROTEINS

Today, plant‐based proteins are a developing market as an alternative to animal‐based ones regarding challenges like population growth and climate changes. However, the plant‐based protein‐rich foods are also growing to meet the nutritional requirement of the public (Jiang et al., 2016). Cereal grains, such as wheat, rye, barley, oats, maize, millet, sorghum, and rice, are considered as a rich source of protein. Cereal proteins as highly accessible plant‐based proteins are balanced in amino acid profile with high digestibility and absorbance in human body (Gong et al., 2020). Consequently, despite the influencing role of proteins in cereal‐based formulations, the incorporation of cereal proteins into other foods is also appealing from nutritional and technological points of view (Loveday, 2019). However, the incorporation of cereal proteins with the aim of improving the nutritional characteristics should also guarantee the acceptability of final product. In other words, no adverse effect on technological characteristics of final products had been achieved via incorporation of cereal proteins.

### 3.1 Technological characteristics of cereal proteins

The technological characteristics of cereal proteins are also considerably important to determine their potential to be applied in food matrices. In this regard, technological characteristics of cereal proteins need to be considered and monitored through food processing to provide the desirable characteristics.

Technological characteristics of cereal proteins as colloidal biopolymers are defined as any physicochemical properties that determine their application in food matrices from a technological point of view. Organoleptic characteristics, surface organization, structural properties, hydration, textural and rheological properties are considered as the main physicochemical properties in the determination of technological functionality of cereal products (Akharume et al., 2021). It has been proven that the technological functionality of proteins is determined by amino acid sequences and their disposition, their flexibility and hydrophobicity, three‐dimensional (3D) conformation, molecular size, net charge, and interaction with other proteins (Corzo‐Martínez et al., 2017). The ability of proteins to be applied efficiently in formulation is generally determined by their solubility, hydrophilic/lipophilic amino acid distribution, the water/oil‐holding capacity, disposition at interface, and consequently the rheological characteristics of its involved media (Martins et al., 2016; Zhu et al., 2017). However, its compatibility with formulation and processing stages also needs to be considered.

Proteins as the main building blocks of food matrices are technologically important besides their nutritional value (Zare et al., 2021). The main technological functionalities of proteins in foods include their ability to form emulsion/foam/gels/film and texturize them (Wang & Xiong, 2019). As cereal proteins comprise more than half of the global food protein supplies, designing and formulation of cereal protein‐based food products seem to be required (Liu et al., 2019). Cereal proteins are used in different formulations with different purposes, for example, as a nonmeat protein alternative (Bohrer, 2019), nondairy yogurt type products (Brückner‐Gühmann et al., 2019), natural protein based food emulsifier (Kim et al., 2020), etc. However, the vital role of cereal proteins in determining the final characteristics of cereal‐derived products like pasta, bread, cake, biscuit, etc., is also non‐negligible. As the direct inclusion of cereal proteins in food products is restricted regarding their generally poor processing characteristics (Malalgoda & Simsek, 2017), using nonthermal to improve treatment has gained increasing attention. However, the technological modification of cereal proteins exposed to nonthermal processing may also be an unwanted outcome of these treatments with the aim of microbial inactivation of cereal.

**FIGURE 2:**
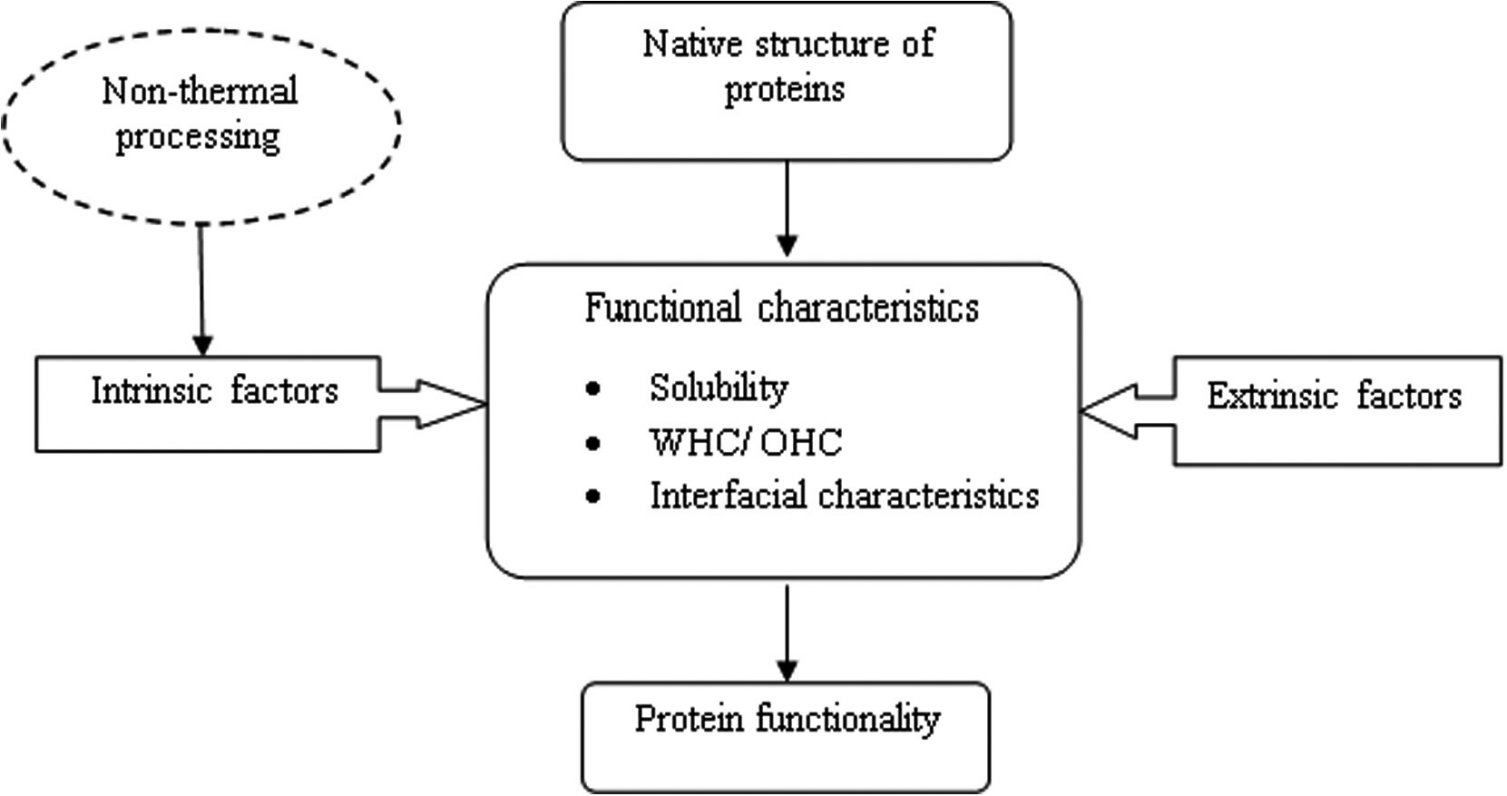
The effect of nonthermal processing on the technological functionality of cereal proteins

Changing the structural, physicochemical, and consequently the technological functionality of protein molecules has been done using different novel nonthermal processings and their combinations. Changes occurred through nonthermal processings that are mainly induced by protein conformational and structural change. In this regard, variation in size, hydration, and surface hydrophobicity will be observed which will then influence the technological functionality and consequently their industrial relevance. As demonstrated in Figure 2, technological functionalities of proteins are influenced by nonthermal processing through intrinsic factors.

Cereal proteins are used for specific function in the formulation and development of food products and as such, understanding the necessary changes in their functionality to improve their potential application (wanted changes) and also their susceptibility to treatments which may be exposed through food product development (unwanted changes) is required. As proteins are complex biomolecules, proposing a general model for their modification is not possible (Mollakhalili‐Meybodi, Yousefi, et al., 2021). In this regard, depending on the functionality modification of interest, considering the state (being in isolation form or embedded in matrix), structural characteristics of the protein as well as the approach to provide a modified functional characteristic seem to be required (Phillips et al., 2011). Considering the nondestructive nature of nonthermal processing, its efficiency in the technological modification of cereal proteins has been reviewed and is summarized in Table 1.

**TABLE 1:** Efficiency of nonthermal processing on technological characteristics of proteins in different cereal types

Treatment	Cereal type	Condition	Finding	Conclusion	References
Irradiation	Wheat	Sample state: Milled wheat Gamma irradiation Dose: 0–30 kGy	Constant protein content (26 ± 0.2%)	Entrapment of radicals produced through irradiation treatment by spatial structure of proteins restricts its adverse effect on protein molecule's size	Jha et al., (2013)
		Sample state: Whole wheat flour (three wheat cultivars as SW‐1, SKW‐355, and HS‐240) Gamma irradiation Doses: 0, 2.5, and 5 kGy	Decrease in water absorption capacity (WAC) from 0.84 to 0.68, 0.83–0.63, and 0.83–0.71 g/g upon irradiation for SKW‐355, HS‐240, and SW‐1, respectively, decrease in oil absorption content from 0.94 to 0.88 and 0.98–0.86 g/g, respectively, for SW‐1 and SKW355, respectively. Decreased emulsion capacity (EC) in a dose‐dependent manner. Increased foaming capacity (FC) and foaming stability (FS) index by increasing irradiation dose	Denaturation of protein and its aggregation decrease its oil absorption capacity (OAC). Decrease in WAC is attributed to the cross‐linking of biopolymers along with the simultaneous chain scission. The decrease in EC might be due to denaturation of proteins and/or protein–protein aggregation upon irradiation. Increased FC and FS may be induced by increased diffusion of unfolded/fragmented proteins toward the air/water interface and higher stability of irradiated protein conformations.	Bhat et al., (2016)
		Sample state: Wheat flour Gamma irradiation Doses: 0, 0.25, 0.5, 1, 5, and 10 kGy	No significant difference in water absorption capacity (WAC), water absorption index (WAI), swelling property, bulk density (either loose or packed), and protein profile via irradiation treatment. The solubility and foaming capacities remain unaffected up to 1 kGy, but significant difference is seen at 5 and 10 kGy. Irregular‐shaped granules at irradiation doses of 5 and 10 kGy by scanning electron microscopy.	Irradiation treatment up to 1 kGy can improve its applicability by providing more flexible proteins.	Manupriya et al., (2020)
		Sample state: Whole wheat flour Gamma irradiation Doses: 0.5, 1, 2.5, 5, and 10 kGy	While the swelling index increased with increasing temperature in control and 0.5 kGy irradiated flour by increasing the temperature, it has been decreased at 90°C for 1 kGy treated and 70°C for 2.5, 5, and 10 kGy irradiated ones.	Protein denaturation resulted in structural modification, to provide hydrophilic groups such as ‐OH, NH2, COOH, and ‐SH.	Bhat et al., (2016)
		Sample state: Gluten samples Gamma irradiation Doses: 1, 3, 6, and 10 Mrad	No significant difference in amino acid profile, except for cystine, which has been decreased at l0 Mrad irradiation dose.	Gel filtration changes revealed the random depolymerization induced by irradiation and configuration changes of gliadin components	Köksel et al., (1998)
		Sample state: Wheat (*Triticum aestivum* L.) Gamma irradiation Doses: 0, 0.5, 1.5, 2.5, and 3.5 KGy	The water absorption capacity (WAC) and degree of softening increased at irradiation doses higher than 0.5 kGy. Irradiation treatment at the dose range of 1.5–3.5 kGy improved the overall bread‐forming capability of wheat flour, which was adversely influenced at doses higher than 3.5 kGy. The extensibility of flour samples has been increased by increasing the irradiation dose	Extensibility of wheat flour determines its gas retention capacity, which is alleviated by irradiation treatment.	El‐Karamany, (2015)
		Sample state: Korean winter wheat variety Keumkang Gamma irradiation Doses: 0, 5, and 10 kGy	Despite no significant difference in protein contents, the dry and wet gluten contents were increased by γ‐irradiation.	Gamma irradiation treatment increased the pasting characteristics of wheat flour and consequently its noodle formation potential.	Lee et al., (2017)
		Sample state: Milled durum samples Gamma irradiation Doses: 0.25, 1, 2.5, 5, and 10 kGy	Despite no significant impact of irradiation treatment on protein content (10.7%–11.0%), the wet gluten has been decreased by increasing the gamma irradiation dose. Higher than 2.5 kGy. No significant difference in sensory perception of lasagna produced from 0.25‐ and 1 kGy‐irradiated semolina has been achieved	Partial damage induced by irradiation treatment at doses higher than 2.5 kGy decreased its wet gluten content and consequently dough stability.	Azzeh & Amr, (2009)
		Sample state: Wheat germ protein hydrolysates (WGPHs) Electron beam irradiation (EBI) Doses: 5, 10, 25, and 50 kGy Voltage: 5 MeV	Increased emulsifying capacity (EC) with its maximum value at 10 kGy irradiated WGPHs. Increased foaming capacity (FC) with its maximum value at 25 kGy.	Maximum EC value of EWGPHs at 10 kGy is attributed to the hydrophilic/hydrophobic balance, which is induced by conformational changes.	Wang et al., (2019)
	Barley	Electron beam irradiation (EBI) Doses: 10, 15, 20, 25, and 30 kGy at room temperature	Despite no significant impact of EBI on protein hydrophobicity at 10 kGy, at doses of 15 kGy and above, a significant increase has been observed by 21%, 35%, 68%, and 80% for irradiation doses of 15, 20, 25, and 30 kGy, respectively.	Breakdown of hydrogen bonds induced by EBI resulted in barley protein conformational changes to expose more hydrophobic sites.	Shawrang et al., (2011)
		Electron beam irradiation (EBI) Doses: 0, 2, 4, 6, 8, and 10 kGy.	Soluble protein has been significantly decreased by 20%.	Protein hydrolysis has been verified by EBI treatment	Kottapalli et al., (2006)
	Millet	Sample state: Millet grain Gamma irradiation Doses: 2, 5, 10, and 15 kGy	Increased protein content (6.85 g/100 g and 8.51 g/100 g in control and 15 kGy irradiated sample). Decreased moisture (12.96 g/100 g and 8.92 g/100 g in control and 15 kGy irradiated sample).	Increased hydration capability of the millet protein fractions exposed to gamma ray increased its moisture content. Dissociation of complex proteins by gamma irradiation increased its protein content.	Reddy & Viswanath, (2019)
		Sample state: Millet grain Gamma irradiation Doses: 0.25, 0.5, 0.75, 1.0, and 2.0 kGy	The protein solubility of millet has been increased from 11.20% to 11.32, 11.82, 13.04, 12.74, and 13.44% in samples irradiated at 0.25, 0.50, 0.75, 1.0, and 2.0 kGy, respectively	The increase in protein solubility after gamma irradiation treatment is induced by high proteolytic activity which resulted in increased hydrolysis of the stored proteins.	Mahmoud et al., (2016)
		Gamma irradiation Doses: 0, 2.0, 4.0, 6.0, and 8.0 kG	No significant change in L*, a*, and b* parameters. The oil absorption capacity (OAC) of millet flour can be influenced only at high doses of gamma irradiation	The OAC is dependent on the potential of physical entrapment of oil through the nonpolar side chains of proteins	Falade & Kolawole, (2013)
	Rice	Sample state: Rice protein Electron beam irradiation (EBI) Doses: 5, 10, 20, or 30 kGy. Voltage: 5 Mev Room temperature	Increased hydrophobicity via electron beam irradiation (EBI). Increased emulsifying abilities of EBI samples in the range of 0.48–0.72. Samples treated by EBI are more vulnerable to environmental conditions. Maximum foaming capacity (FC) is observed by 10 kGy irradiated samples	EBI treatment unfolded the rice proteins with its degree increased by increasing the irradiation dose. The conformational changes resulted in a decrease in α‐helices and increase in β‐sheets, β‐turns, and random coils. The increased hydrophobicity induced by EBI decreased the emulsion stability index, which is expected to increase oil droplets’ flocculation. The EBI treatment resulted in protein scission and conformational changes.	Zhang, Wang, Chen, et al., (2019)
		Sample state: Rice seeds Gamma irradiation Doses: 0.01, 0.1, and 1 kGy	Decreased firmness by treatment	Cross‐links induced by gamma irradiation decrease the curd firmness	Sung, (2005)
	Sorghum	Sample state: Whole sorghum flour Gamma irradiation Doses: 0, 10, and 50 kGy	No significant difference has been found in the nitrogen solubility index at 10 kGy irradiation treatment but it significantly decreased at an irradiation dose of 50 kGy in wet flour.	The enhanced influence of irradiation treatment in wet flour is induced by the reactions of products of water radiolysis with protein molecules	Fombang et al., (2005)
		Sample state: Sorghum seeds Electron beam irradiation (EBI) Doses: 10, 15, 20, 25, and 30 kGy	The protein content has been significantly increased at doses higher than 15 kGy	The potential of EBI to reduce antinutritional factors and increase the protein digestibility of sorghum protein	Shawrang et al., (2011)
		Sample state: Sorghum grain Gamma irradiation Doses: 0.5, 1, 2, 3, 4, and 5 kGy	Increasing the gamma irradiation dose significantly decreases the emulsifying and increases the emulsifying activity (EA). However, EA of the flour significantly are also decreasing at doses higher than 1 kGy. However, no significant changes had been found in foaming capacity (FC) in irradiation doses up to 2.0 kGy, and it has been decreased at doses higher than 2 kGy.	Gamma irradiation at low doses can be considered as a safe method for elimination of the fungal incidence in stored sorghum grains.	Ahmed et al., (2018)
Cold plasma (CP)	Wheat	Sample state: Milled flour (hard and soft wheat) Plasma source: Radio‐frequency (RF) Gas types: Argon and carbon dioxide Flow rates: 10 and 25 cm^3^/min	No significant difference in protein solubility. While the proportion of *β*‐turns in soft wheat has been increased by plasma treatment, the β‐sheets’ proportion decreased. The extensibility of soft wheat has been decreased.	The lower β‐sheets in irradiated soft wheat mean its lower ability to be aggregated and consequently impaired network formation to hold produced gases.	Held et al., (2019)
		Sample state: Wheat flour (*Triticum aestivum* L.) Treatment times: 60 s, 120 s Input voltages: 15 and 20 V. Input gas: Surrounding air	No significant difference in the total protein content with higher molecular weight at the highest treatment (20 V for 120 s).	Increasing the molecular weight by increasing the treatment intensity verifies the increased strength of its derived dough.	Bahrami et al., (2016)
		Sample state: Wheat seed Plasma source: DBD (dielectric barrier discharge) Voltages: 0.0, 9.0, 11.0, 13.0, 15.0, and 17.0 kV (CK, T1, T2, T3, T4, and T5, respectively) Gas flow: 1.5 L.min^−1^ Time: 4 min Gas type: Dry air	Increasing the voltage content increased the soluble protein content from 30.9 mg/g to 34.1 and 35.6 mg/gin T2 and T3 samples, respectively, which were again decreased at T4 and T5 with 33.3 and 31.4 mg g^−1^, respectively.	DBD plasma treatment is considered as an efficient approach to improve wheat seed functionality	Guo et al., (2018)
		Sample state: Wheat grains (*Triticum aestivum* L.) and wheat flour Plasma source: DBD (dielectric barrier discharge) Voltage: 80 kV Treatment times: 5, 10, 20 and 30 min will be termed as PTF‐5, PTF‐10, PTF‐20, and PTF‐30. Gas type: air	Increasing the treatment time enhances the oil holding capacity (OHC) of PTF from 0.80 ± 0.036 g/g in control and 0.86 ± 0.08 g/g after 30 min plasma treatment. No significant difference in WBC has been observed.	No significant changes have been observed in protein structure and conformation via cold plasma treatment regarding Fourier transform infrared (FTIR) spectra.	Chaple et al., (2020)
		Sample state: Wheat seeds Plasma source: DBD (dielectric barrier discharge) Voltage: 0–50 kV Frequency: 50 Hz Plasma source: DBD Gas sources: Argon (Ar), oxygen (O_2_), nitrogen (N_2_), and air	No significant difference has been observed between the soluble protein content of control and O_2_ plasma treated samples. It was increased to from 30.9 to 35.6, 38.8, and 40.7 mg/g for the air, N_2_, and Ar plasma treatments. After the DBD plasma treatment for 4 min, the highest soluble protein content has been observed in Ar plasma treated samples.	Enhanced seed germination after DBD plasma treatment increased its soluble protein content.	Meng et al., (2017)
	Barley	Sample state: barley grain Plasma source: DBD (dielectric barrier discharge) Gas type: air Treatment times: 0, 2, 4, 6, 8, and 10 min Current: 1 A Power: 300 W Frequency: 3500 Hz	No significant change in protein content (10.68% in control sample and 10.26 after 10 min cold plasma treatment)	No significant changes had been found in quality parameters of barley after cold plasma treatment.	Feizollahi et al., (2020)
	Brown rice	Sample state: Brown rice seed Plasma source: Radio‐frequency (RF) Gas type: air Relative humidity: 45.3 ± 0.3% Voltages: 1500 V and 1750 V	No significant difference in protein content	No significant difference has been found in protein content	Thirumdas et al., (2016)
		Sample state: Parboiled rice Plasma source: Radio‐frequency (RF) Gas type: Power: 30, 40, and 50 W Times: 5, 10, and 15 min	Increased protein content from 5.97 to 6.03–6.18 depending on time and power.	The increase in protein content is through disintegration of surface proteins and proteinaceous matters induced by atomic oxygen.	Sarangapani et al., (2015)
Pulsed electric field (PEF)	Wheat	Sample state: Gliadin protein Electric field intensity of 10 kV/cm and 20 kV/cm	No significant difference in the solvent accessible surface area as an indicator of protein's potential to interact with other molecules. No distinct alpha helices and beta sheets have been found and the only visible structure was the turns	Verified conformational changes with no significant effect on the protein surface	Singh et al., (2013)
		Sample state: Gluten concentrate suspension (pH = 5, 6 at 5% w/w) Electric field strength (E = 1.65 kV/cm)	Increased solubility, water holding capacity (WHC), and oil holding capacity (OHC)	Using PEF at pH lower than its isoelectric point enhances the exposure of protein charge and increases the solubility of proteins	Melchior et al., (2020)
	Oat	Voltage: 2.0–2.2 kV/cm and 4.0–4.4 kV/cm Pulse energy: 1.2–9.7 J, combined with three specific energy input levels (range of 48–53 kJ/kg, 200–249 kJ/kg, and 418–484 kJ/kg)	Changes in the intensity of 1635 and 1650 cm^−1^ peaks of Fourier transform infrared (FTIR) spectra have been found for both raw and thermally processed oat flours	Modification in the secondary structure of both flour proteins has been observed. PEF can change the β‐sheets to α‐helix structure of proteins to promote the protein molecules unfolding, which is necessary to determine the oat protein's technological functionality.	Duque et al., (2020)
	Oat	Pressure: 200, 300, 350, 400, or 500 MPa. S Time: 10 min	The oat proteins had been significantly influenced by high hydrostatic pressure (HHP). Improved viscosity and elasticity have been observed in HHP‐treated samples. Despite the dominance of viscose to elastic model, at 300 MPa treated samples, the reverse is observed in samples treated at higher pressure. The amount of water/salt‐soluble fraction (at 350 MPa) and the amount of urea‐soluble proteins (at 300 MPa) have been decreased	Decrease in solubility verifies the formation of disulfide bond. However, HHP‐induced denaturation, gelation, aggregation, and/or enhanced interaction with other components can also be considered	Hüttner et al., (2009)
	Rice	Rice suspension in phosphate buffer (pH=5, 6 at 5% w/w) Electric field strength (E = 1.65 kV/cm)	No significant difference in protein aggregation and its primary structure. Increase in sulfhydryl groups. Decreased solubility, regardless of pH	Partial protein unfolding has been revealed.	Melchior et al., (2020)
Ultrasonication (US)	Barley	2.5 min ultrasound treatment, cooling and another 2.5 min ultrasound treatment. Temperature was kept lower than 35°C.	The protein solubility and colloidal stability of barley‐derived proteins at alkaline pH have been improved and the particle size at all pH values has been decreased	As no significant changes have been found in protein profiles through US, it can be considered as a promising tool for alleviating the applicability of barley protein in liquid food formulation	Silventoinen & Sozer, (2020)
	Millet	Sample state: Millet protein concentrate (MPC) dispersion (10% w/w) Sonication times: 5, 12.5, and 20 min Amplitudes: 20%, 60%, and 100% with constant pulse durations	The solubility of MPC has not been significantly influenced by increasing the US time from 5 to 20 min at 18.4 W/cm^2^ intensity. At 73.95 W/cm^2^ intensity, the solubility has been decreased by increasing the US time from 12.5 to 20 min. A decrease in foaming capacity and no improvement in foaming stability at low intensities and times of US treatment have been observed.	Conformational changes through ultrasonic treatment to affect its surface hydrophobicity have been revealed.	Nazari et al., (2018)
	Rice	Sample state: Rice dreg (75.51% protein) Two intervals of ultrasound frequency (20 + 28, 20 + 35, 20 + 40, 20 + 50 kHz working as pulsed‐on 10 s and ‐off 5 s).	Using US significantly decreased the lysinoalanine content by 12.2% in 20/40 kHz treated sample,	Decrease in amino acids like threonine, lysine, and arginine induced by sonication will reduce the formation of lysinoalanine.	Zhang et al., (2020a, 2020b)
		Sample state: Rice dreg mono‐frequency ultrasound (MFU), dual‐frequency ultrasound (DFU), and tri‐frequency ultrasound (TFU)	Ultrasound pretreatment resulted in conformational change by decreasing all the ultrasound pretreatment *α*‐helix, *β*‐turn and increasing *β*‐sheet, random coil at all frequencies and working modes.	No significant difference in the degree of hydrolysis of protein has been observed.	Yang et al., (2017)
High hydrostatic pressure (HHP)	Wheat	Sample state: Wheat flour suspensions (40% w/w) Pressure: 200, 300, 400, 500, or 600 MPa Temperature: 20°C	Decreased complex modulus at 200 MPa pressure.	Low hydrostatic pressure resulted in depolymerization of proteins	Vallons et al., (2010)
	Oat/millet/sorghum	Sample state: Hydrated oat/millet/sorghum flours Time: 10 min Temperature: 20°C Pressure: 350 MPa	No significant difference in physicochemical characteristics and improved nutritional/sensory profiles of HHP‐treated breads	Improved digestibility of proteins is attributed to its conformational changes through HHP treatment and inactivation of protease inhibitors.	Angioloni & Collar, (2012)

### 3.2 Nutritional characteristics of cereal proteins

As noted in previous sections, one of the main benefits of nonthermal technologies is to ensure the safety of food with minimal damages or even improving the nutritional quality (Ekezie et al., 2017). The effect of these methods on the nutritional properties of proteins in cereal products with respect to changes in allergenicity, bioavailability, digestibility as well as amino acids and proteins content will be discussed in the following. Table 2 shows different studies that investigated the effects of nonthermal technologies on the nutritional properties of different cereals and their products.

**TABLE 2:** The effects of nonthermal methods on the nutritional properties of cereal proteins

Treatment	Application	Cereal type	Condition	Finding	Conclusion	Reference
Irradiation	Digestibility	Rice	Sample state: Rice grains (*Oryza* *sativa)*	Conformational changes of proteins	No significant effect on easy‐to‐digest and difficult‐to‐digest proteins	(Maity et al., 2009)
			Gamma irradiation	Increase in protein hydrophobicity		
			Doses: Range of 1–6 kGy (0.12 kGy/h) at 25°C			
		Millet and Sorghum	Sample state: Sorghum, pearl millet, foxtail millet (Whole and dehulled grains)	Changes the protein structure	Improvement in in vitro protein digestibility (3.1%–5.0%)	(Sujatha et al., 2017)
			Gamma irradiation	Cleavage of disulfide bond proteins		
			Doses: 1.0 kGy and 2.5 kGy			
		Barley	Sample state: Barley grains	Increased denaturation of proteins	Increase in protein digestibility dependent on increased radiation dose	(Parvin Shawrang et al., 2013)
			Electron beam radiation (EBI)	Increased hydrophobicity of protein (3%–80%)		
			Doses: 10, 15, 20, 25, and 30 kGy at room temperature			
	Amino acid contact	Wheat, Barley, Corn, Sorghum	Sample state: Cereal grains	Hypersensitivity of sulfur amino acids to radiation	Decrease in methionine	(Aziz et al., 2006)
			Gamma irradiation			
			Doses: 1, 3, 5, 10, and 15 kGy (4 kGy/h) at room temperature			
		Rice	Sample state: Rice proteins	Increased proteolysis	Increase in essential amino acids	(Zhang et al., 2020a, 2020b)
			Electron beam irradiation (EBI)	Hypersensitivity of sulfur amino acids to radiation	Decrease in methionine and cystine	
			Doses: 0, 5, 10, 20, and 30 kGy at room temperature			
	Bioactivity	Rice	Rice proteins	Induction protein unfolding	Increase in antioxidant activity	(Zhang et al., 2020a, 2020b)
			Electron beam irradiation (EBI)	Promotion of hydrolytic efficiency of polypeptides		
			Doses: 5, 10, 20, and 30 kGy	Production of low‐molecular‐weight peptides		
				Increase in hydrophobic amino acids (HAA)		
		Corn	Corn gluten meal	Increase in the degree of hydrolysis of proteins	Increase in antioxidant activities	(Lin et al., 2014)
			Electron beam irradiation (EBI)			

			Doses: 0, 1.08, 2.16, 3.24, 4.32, 5.4, and 6.48 kGy (1.08 kGy/s)			
High hydrostatic pressure (HHP)	Allergy	Rice	Rice grains	Changes in endosporium membrane permeability	Decrease in allergenic proteins	(Kato et al., 2000)
			Pressure: 100–400 MPa at 20°C	Changes in the structure of 16 kDa albumin, α‐globulin, and 33 kDa globulin and their release into the surrounding solution		
			Time: 30 min			
	Bioactivity	Rice	Soaked milled rice grains	Increased denaturation of proteins	Increase in gamma‐aminobutyric acid (GABA) (as a bioactive compound)	(Yamakura et al., 2005)
			Pressure: 400 Mpa	Increased proteolysis		
			Time: 10 min			
	Amino acid contact	Rice	Soaked milled rice grains	Increased denaturation of proteins	Increase in some free amino acids	(Yamakura et al., 2005)
			Pressure: 400 Mpa	Increased proteolysis		
			Time: 10 min			
Ultrasound	Bioactivity	Rice	Rice dreg	Loosening of the protein structure	Increase in angiotensin‐I‐converting enzyme (ACE) inhibitory activity peptides	(Yang et al., 2017)
			Mono‐frequency ultrasound (MFU), dual‐frequency ultrasound (DFU), and tri‐frequency ultrasound (TFU)	Decrease in α‐helix content		
				Increase in β‐sheet		
Pulsed electric field (PEF)	Bioactivity	Corn	Corn peptides (10–30 kDa)	Changes in the structure of peptides	Increase in antioxidant activities	(K. Wang et al., 2015)
			Intensity: 15 kV/cm, pulse frequency 2,000 Hz			
			(15 kV/cm)			
			2000 Hz)			
Cold plasma (CP)	Allergy	Wheat	Wheat proteins	Changes in the structure of wheat allergenic proteins	Reduction of wheat allergen potency	(Nooji, 2011)
			Plasma source:	Decrease in immunoglobulin E (IgE) binding		
			frequency of 60 Hz			
			(30 kV, 5 min)			
Pulsed light (PL)	Allergy	Wheat	Wheat gluten	Alteration in the structure or destruction of gliadin epitopes	Reduction of allergenicity of wheat gluten	(Panozzo et al., 2016)
			Pulsed light:			
			1.75–26.25 J/cm^2^			

Food allergy is one of the global problems affecting almost 10% of the world's population with its incidence being twice in the children (Rodrigues et al., 2020). Food allergy is defined as an abnormal immune system response (immunoglobulin E (IgE)) to antigens that commonly include low‐molecular‐weight (10–70 kDa) proteins or water‐soluble glycoproteins (Sarangapani et al., 2018). Generally, more than 170 types of allergenic foods have been investigated, of which 90% of them related to allergenic proteins are found in foods, such as wheat (Ekezie et al., 2017). Purification of wheat flour water‐soluble proteins confirmed the presence of 27 allergenic proteins, especially prolamine, in this product (Guerrieri & Cavaletto, 2018).

Recently, it has been demonstrated that nonthermal food processing has the potential to reduce food allergens (López‐Pedrouso et al., 2019; Rodrigues et al., 2020). The effect of HHP method on allergen proteins has been studied and it was illustrated that high pressures by three main mechanisms can inhibit the allergenicity of proteins: (i) Effect on cell wall permeability or damage it, followed by release and removal of allergen proteins (Kato et al., 2000). (ii) Breakdown of allergen proteins into smaller subunits without allergenic properties (Zhou et al., 2016). (iii) Alterations in the structure of these proteins, consequence of deformation, and inactivation of epitopes (Somkuti & Smeller, 2013). For instance, high pressures of up to 300 MPa showed the ability to partially destroy the endosporium cell membrane in rice grains, while at higher pressures this damage increased and led to activate solubilization and release of allergenic proteins in rice. Also, using HHP method with protease solutions caused an improvement of high pressure performance in the release of allergenic proteins due to increased permeability of the endosporium cell membrane to protease solutions (Estrada‐Girón et al., 2005). The results of applying CP on wheat proteins have also determined a 37% reduction in the allergenicity of wheat proteins (Nooji, 2011). Although the mechanism of this reduction effect is not yet fully understood, it is hypothesized that the reactive species in CP have the ability to disrupt or mask linear epitopes of proteins, making them unrecognizable by immunoglobulin (Sarangapani et al., 2018).

Bioactive peptides are low‐molecular‐weight proteins containing 2–23 amino acids that have health‐promoting functions, such as cholesterol‐lowering, anticancer, antihypertensive, and anti‐inflammatory effects (Rodrigues et al., 2020). Some proteins potentially have bioactive dipeptides in their polypeptide structure that can be released during fermentation, enzymatic hydrolysis, processing, and digestion (Rodrigues et al., 2020). Although milk and egg proteins have the highest potential to be converted to bioactive peptides, plant proteins, including cereals, also have the ability to produce these compounds (Tsevdou et al., 2019). The results of many studies indicated that partial denaturation of proteins using high pressures resulted in easier access for hydrolyzing enzymes to the protein cleavage sites which could increase bioactive peptides (Tsevdou et al., 2019). In this regard, it is revealed that applying pressures up to 100 MPa could increase the production of free amino acids and gamma‐aminobutyric acid (GABA) bioactive compound in germinated rough rice (Kim et al., 2015). It is believed that protease activity increasing and consequently glutamine levels’ elevation may be the reason for the increase in GABA (Chua et al., 2019). Investigations have confirmed the effect of radiation on increasing the degree of hydrolysis of proteins and subsequently, production of low‐molecular‐weight peptides, which have antioxidant activity due to their ability to react with free electrons (Wang et al., 2019; Zhang et al., 2020a, 2020b). In fact, the availability of hydrophobic groups in small peptide fragments and proteins makes them have more potential to eliminate free radicals (Zhang et al., 2020a, 2020b). In a study on the low‐molecular‐weight proteins of corn peptides, it was investigated that the PEF treatment could improve the antioxidant activity of these proteins by increasing the solubility of proteins through polarizing (Wang et al., 2015).

Given the importance of proteins in the diet, their digestibility has become one of the most interesting topics in the food industry. The digestibility of proteins is mainly influenced by external and internal factors. Internal factors are related to the structure and bonds within the protein, while external factors are related to other environmental compounds such as antinutrient compounds (Joye, 2019). Antinutritional compounds are known as deleterious compounds that interfere with the digestibility and bioavailability of nutrients (Mollakhalili‐Meybodi et al., 2021). In cereals, antinutrient agents like phytates have the ability to reduce the digestibility of proteins (Nikmaram et al., 2017). In addition, some compounds such as trypsin and chymotrypsin inhibitors which are also protein in nature could prevent the digestibility and absorption of proteins and other nutrients (Kostekli & Karakaya, 2017).

It is expected that nonthermal methods could cause easier access of hydrolytic enzymes to the peptide chain through denaturation of proteins and subsequently increase their digestibility. However, there are conflicting reports regarding the effect of these processings on the digestibility of cereal proteins. For example, irradiation increased the digestibility of corn, while it decreased that in the sorghum flour proteins (Hassan et al., 2009) and had no effect on the digestibility of rice grain proteins (Maity et al., 2009). Also, the use of high pressures in the production of breads made from oat, sorghum, or millet flours reduced the digestibility of proteins (Angioloni & Collar, 2012). Therefore, it can be concluded that the effect of these methods depends considerably on the protein structure of cereals. For instance, sorghum has naturally lower digestibility than other grains due to the high amount of disulfide bonds in its protein structure (Annor et al., 2017). Also, owing to the denaturation of proteins and exposure of hydrophobic regions, proteins’ aggregation may occur which could reduce their digestibility (Gulati et al., 2017).

Most studies showed that there were no significant changes in the quantity of proteins under the application of nonthermal methods. For example, it has been illustrated that the gamma radiation has no or minimum significant effect on the levels of proteins and amino acids (Lee & Kim, 2018). In contrast, several studies have shown a decrease in the amounts of amino acids in these treatments. The most important reasons for this decline could be changes in the solubility of amino acids and their possible excretion from food, increased sensitivity of amino acids to reactions such as oxidation, decomposition of complex amino acids, and their conversion into simpler amino acids (Tolouie et al., 2018). The irradiation of rice proteins has also revealed that the levels of the methionine and cysteine decreased significantly with increasing radiation dose due to their sensitivity to oxidation (Zhang et al., 2020a, 2020b). High pressures showed dissimilar effects on amino acid contents. In this regard, it was found that HPP treatment significantly increased amino acid contents in the brown rice by increasing proteolytic activity, with the exception of glutamine due to its possible conversion to other compounds by certain metabolic pathways (Shigematsu et al., 2010).

## 4 CONCLUSION

Considering the importance of the technological and nutritional characteristics of cereal proteins, their monitoring through processing seems to be necessary. Despite the nondestructive nature of nonthermal processing, their potential impact on the structure (quaternary, tertiary, secondary, and primary) of cereal proteins may influence their performance in food matrices from both technological and nutritional perspectives. The impacts of nonthermal processing on technological characteristics of cereal proteins may be achieved either purposefully or unintended. In other words, despite the changes induced by nonthermal processing to improve its applicability in food matrices, its impact on protein structure as a microbial inactivation treatment is unwanted. Nutritionally, the influence of nonthermal processing on allergenicity, bioavailability, digestibility, and amino acid profile is considered to be important. The main mechanism of nonthermal processing treatment on cereal proteins is achieved by its impact on protein structure and consequently the conformational changes. In this regard, choosing an appropriate treatment for cereal proteins to keep/improve their technological and nutritional performance considering their potential application is recommended.

## CONFLICTS OF INTEREST

The authors declare that they have no known competing financial interests or personal relationships that could have appeared to influence the work reported in this paper.

## ETHICS APPROVAL

This study has been ethically approved, IR.FUMS.REC.1400.010.

## Data Availability

The datasets used and/or analyzed during the current study are available from the corresponding author on reasonable request.
